# The effects of repetitive transcranial magnetic and transcranial direct current stimulation on memory functions in older adults with mild cognitive impairment: a systematic review and meta-analysis

**DOI:** 10.3389/fnhum.2024.1436448

**Published:** 2024-08-13

**Authors:** Mengdie Hu, Michael A. Nitsche, Yanxin Lv, Hairong Han, Xu Lin, Fengxue Qi

**Affiliations:** ^1^School of Sports Medicine and Rehabilitation, Beijing Sport University, Beijing, China; ^2^Department of Psychology and Neurosciences, Leibniz Research Center for Working Environment and Human Factors, Dortmund, Germany; ^3^University Clinic of Psychiatry and Psychotherapy, Protestant Hospital of Bethel Foundation, University Hospital OWL, Bielefeld University, Bielefeld, Germany; ^4^German Center for Mental Health (DZPG), Bochum, Germany; ^5^Sports, Exercise and Brain Sciences Laboratory, Sports Coaching College, Beijing Sport University, Beijing, China; ^6^Blood Purification Department, The Eighth People’s Hospital of Jinan, Shandong, China; ^7^Shandong Mental Health Center, Shandong University, Jinan, China

**Keywords:** mild cognitive impairment, memory, repetitive transcranial magnetic stimulation, transcranial direct current stimulation, cognitive function, non-invasive brain stimulation

## Abstract

Mild cognitive impairment (MCI) is a condition that impairs activities of daily living, and often transforms to dementia. Repetitive transcranial magnetic stimulation (rTMS) and transcranial direct current stimulation (tDCS) show promise in improving cognitive functions in MCI patients. In this meta-analysis, we aimed to compare the effects of rTMS and tDCS on memory functions in MCI patients. We explored eight databases from their inception to March 16, 2024. We obtained 11 studies with 406 patients with MCI. We used the standardized mean difference (SMD) with a 95% confidence interval (CI) to synthesize the effect size. rTMS and tDCS significantly improved memory functions in MCI patients (SMD = 0.61; 95% CI: 0.41–0.82; *p* < 0.00001; I^2^ = 22%). In subgroup analysis of number of stimulation sessions, both rTMS and tDCS over 10 sessions (SMD = 0.84; 95% CI: 0.50–1.17, *p* < 0.00001, I^2^ = 0%) significantly improved the memory function in MCI patients. The subgroup analyses on different stimulation types (SMD = 0.78; 95% CI: 0.51–1.06; *p* < 0.00001; I^2^ = 0%) and treatment persistent effects (SMD = 0.93; 95% CI: 0.51–1.35, *p* < 0.0001, I^2^ = 0%) showed that rTMS was more effective than tDCS. rTMS with a stimulation frequency of 10 Hz (SMD = 0.86; 95% CI: 0.51–1.21; *p* < 0.00001; I^2^ = 0%) and over 10 sessions (SMD = 0.98; 95% CI: 0.58–1.38; *p* < 0.00001; I^2^ = 0%) at multiple sites (SMD = 0.97; 95% CI: 0.44–1.49; *p* = 0.0003; I^2^ = 0%) showed a great improvement in the memory performance of patients with MCI. rTMS was more likely to appear temporary side effects (risk ratio (RR) = 3.18, 95% CI: 1.29–7.83, *p* = 0.01). This meta-analysis suggests that rTMS and tDCS are safe and efficient tools to improve memory functions in patients with MCI, while rTMS had a larger effect than tDCS. rTMS with a stimulation frequency of 10 Hz targeted on multiple sites over 10 sessions showed the greatest effect. We could not conclude parameters of tDCS because of insufficient data.

**Systematic Review Registration:**

https://www.crd.york.ac.uk/prospero/display_record.php?ID=CRD42024558991.

## 1 Introduction

Mild cognitive impairment (MCI) is a condition that impairs activities of daily living due to cognitive decline and often develops into full dementia (Petersen, 2004; Jack et al., 2011). Dependent on the involvement of memory impairment, it is divided into amnestic MCI and non-amnestic MCI (Petersen, 2016). Patients with MCI may show alterations of brain structure and function (Pereira et al., 2018). Here, the medial temporal lobe shows the earliest structural changes, including the hippocampus, parahippocampal, perirhinal, and entorhinal regions (Oedekoven et al., 2015). Memory functions, especially associative memory and episodic memory, are reduced primarily due to changes in the hippocampus, which is a part of the medial temporal lobe, and crucial for encoding and retrieving events (Diana et al., 2007; Dickerson and Eichenbaum, 2010). Functional neuroimaging studies revealed that the decline of memory performance in MCI patients is related to the reduced hippocampal activation (Oedekoven et al., 2015). At present, clinical data show that no effective pharmacological treatments to improve cognitive impairment are available (Petersen et al., 2018). Therefore, alternative non-pharmacological interventions to treat MCI are probed.

Non-invasive brain stimulation is a technique used to induce neuronal plasticity of the brain by modulating excitability of cortical neurons, and includes repetitive transcranial magnetic stimulation (rTMS) and transcranial direct current stimulation (tDCS) (Teselink et al., 2021). In recent years, non-invasive brain stimulation has been probed for the treatment of neuropsychiatric disorders and appears to be a promising treatment for ameliorating cognitive impairment. rTMS induces alterations of cerebral excitability for a period that outlasts the intervention by inducing electrical pulses to the brain via repetitive magnetic pulses applied at regular intervals to the scalp (Klomjai et al., 2015). It alters the excitability of nerve cells via suprathreshold electrical stimuli induced in the brain, which activate neurons. Repetitive application of these stimuli induces synaptic plasticity. According to the stimulation frequency, it can be divided into high-frequency rTMS (≥ 5 Hz) and low-frequency rTMS (≤ 1 Hz) (Pascual-Leone et al., 1998). A novel rTMS protocol, namely theta burst stimulation (TBS), applies magnetic stimuli in bursts of three pulses at 50 Hz with an interval of 5 Hz (Huang et al., 2005), and is applied in two different patterns: intermittent theta burst stimulation (iTBS), which produces facilitatory effects on cortical excitability, and continuous theta burst stimulation (cTBS), which reduces cortical excitability (Huang et al., 2005). The mechanisms of the after-effects of rTMS and TBS are suggested to be similar to long-term potentiation (LTP) and long-term depression (LTD). LTP and LTD were first developed in animal models and reflect changes of synaptic strength induced by high-frequency, or low frequency stimulation. LTP is defined as an increase, while LTD reflects a decrease of synaptic strength (Klomjai et al., 2015). The frequency of stimulation determines the induction of LTP or LTD. High-frequency rTMS and iTBS have excitatory effects leading to LTP, while low-frequency rTMS and cTBS induce LTD (Nabavi et al., 2014; Klomjai et al., 2015). There is strong evidence experiment in rats showing that LTP and LTD play important roles in learning and memory (Nabavi et al., 2014; Connor and Wang, 2016). Some studies have shown that rTMS focused to certain brain regions, such as the precuneus, improve cognitive functions in MCI patients, which may make a reduction in excessive functional compensation to protect cortical plasticity of cerebrum (Ge et al., 2023). Furthermore, high-frequency rTMS targeted over the left dorsolateral prefrontal cortex (DLPFC) has been reported to improve memory functions in patients with MCI, which is suggested to be due to its interaction with the medial temporal network, contributing to executive and memory functions (Blumenfeld and Ranganath, 2006; Blumenfeld et al., 2011; Chou et al., 2020).

tDCS is a non-invasive brain stimulation method using low direct currents (1–2 mA) applied across the cortex using two or more electrodes (Chase et al., 2020). The mechanism of tDCS is thought to be an alteration of cortical excitability through modulation of resting membrane potentials. Anodal stimulus increases excitability and cathodal stimulus decreases excitability of the targeted areas when using conventional protocols, and sufficiently long-lasting intervention protocols result in neuroplastic after-effects (Brunoni et al., 2012). Anodal tDCS with conventional protocols increases cortical excitability, and induces LTP-like plasticity, while cathodal tDCS diminishes excitability, and generates LTD-like plasticity (Kronberg et al., 2017). Numerous studies reported that tDCS effectively improved cognitive abilities, such as memory and attention (Chase et al., 2020).

A few meta-analyses have explored the effect of rTMS in MCI patients. One meta-analysis focused on cognition of MCI patients treated by rTMS, including subgroup analyses of global cognition, memory, stimulation sites, and number of stimulation sessions (Zhang et al., 2021). Their results indicated that with high frequency, larger stimulation sessions, and multiple sites, rTMS brought about a greater improvement in cognition in MCI patients. Another meta-analysis explored the impact of rTMS alone or rTMS combined with pharmacological treatment on global cognition, episodic memory, and verbal fluency in MCI patients (Zhou et al., 2020). Compared to sham stimulation, rTMS produced improvement in global cognition, episodic memory, and verbal fluency in MCI patients. However, there was no significant difference in memory quotient compare rTMS plus pharmacological therapy to pharmacological treatment alone. There were also meta-analyses focused on the effects of tDCS in patients with MCI. For example, Cruz Gonzalez et al. explored whether tDCS alone or combined with cognitive training could improve cognitive functions in MCI and dementia patients. And they found that overall, tDCS alone achieved significant improvement in memory of MCI patients (Cruz Gonzalez et al., 2018). Talar et al. (2022) did meta-analysis on the effects of aerobic exercise paired with tDCS, aerobic exercise alone and tDCS alone in global cognition, working memory and executive function in healthy older adults, MCI and dementia patients. The results showed that tDCS had no effects on the three cognitive outcomes in patients with MCI. Although rTMS and tDCS have been used to treat patients with MCI, a comparative exploration of the effects of both methods is so far missing. Based on the existence evidence, there was no comparation on the effects of rTMS and tDCS without other interventions on memory functions in MCI alone. And to our knowledge there was no article exploring the parameters such as stimulation regions, number of stimulation sessions, frequencies and intensities of rTMS and/or tDCS in memory functions of MCI alone. It is essential to provide better treatment suggestions for clinical practitioners to treat the MCI patients, we included newly studies with strict inclusion criteria to evaluate the best treatment therapy and proper parameters. We aimed to close this gap by exploring the memory effects, and side effects of rTMS and tDCS in patients with MCI.

## 2 Materials and methods

### 2.1 Search strategy

**Figure 1:**
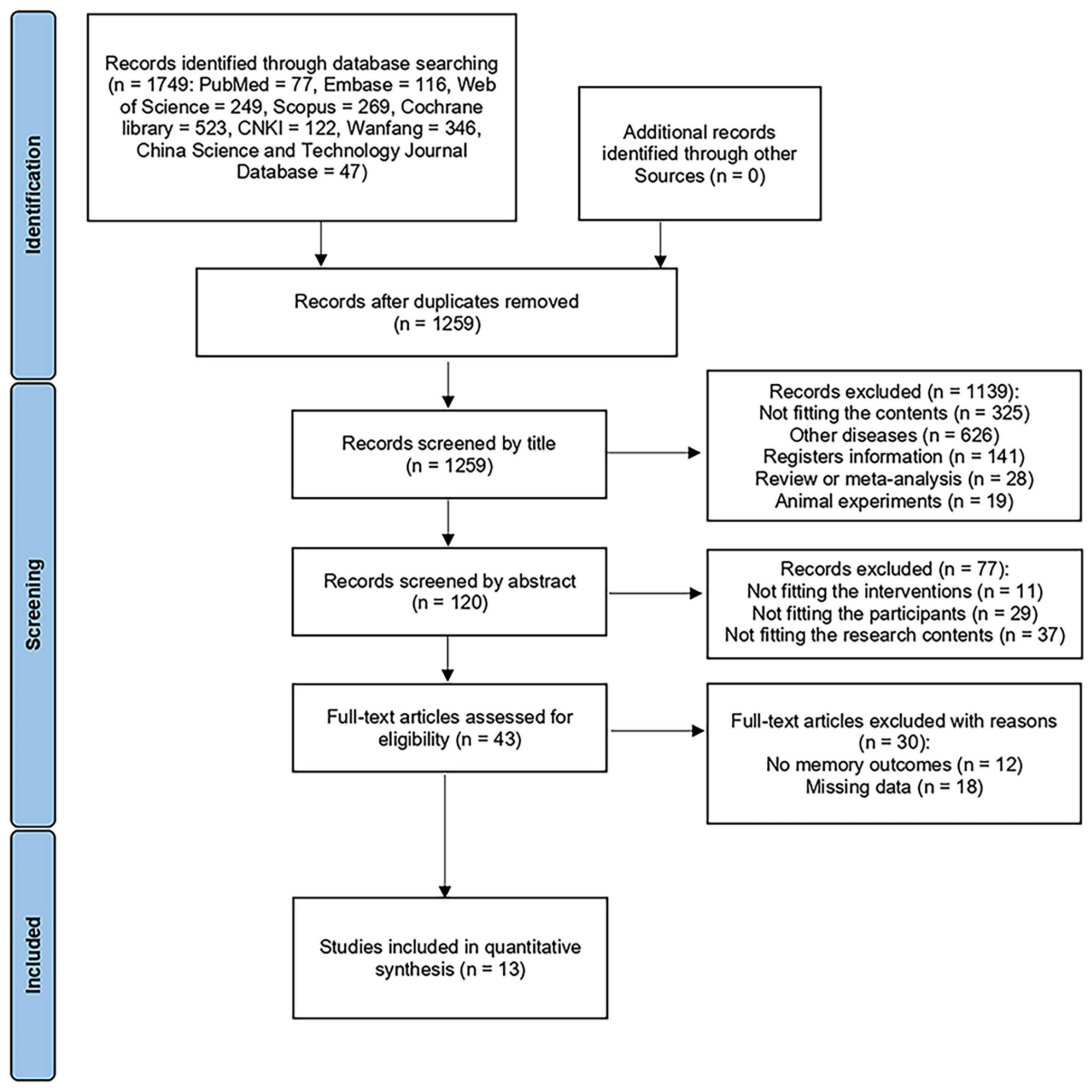
The flowchart of search procedure.

The Preferred Reporting Items for Systematic Review and Meta-Analyses (PRISMA) guidelines were followed in this study. We conducted this systematic review and meta-analysis by searching suited studies in eight databases from their inception to March 16, 2024, including PubMed, Embase, Web of Science, Scopus, the Cochrane Library, the China National Knowledge Infrastructure, Wanfang, and the China Science and Technology Journal Database. To ensure that respective studies were extracted accurately, two independent authors were involved in the assessment of relevant articles. Any disagreements were resolved by discussions with a third arbitrator and a consensual decision. The search process is illustrated in Figure 1. We retrospectively registered the protocol of this meta-analysis at PROSPERO (No. CRD42024558991), with the date of registration 29/6/2024.

### 2.2 Inclusion and exclusion criteria

In accordance with the PICOS (participants, interventions, comparison, outcome and study design) criteria, studies that fulfill all of the following criteria were included: (1) participants ranged in age from 50 to 90; (2) participants were diagnosed with amnestic MCI or non-amnestic MCI by neurologists, or met the Petersen’s criteria (Petersen, 2004) or other criteria (clinical/neuropsychological criteria, Mayo Clinic Criteria, Guidelines for Diagnosis and treatment of dementia and cognitive disorders in China, and the criteria of the MCI Working Group of the European Consortium on Alzheimer’s disease); (3) non-invasive brain stimulation was used as intervention in the experimental group; (4) outcomes included memory; (5) randomized controlled trial as the trial design.

Articles were excluded when met one of following criteria: (1) meta-analyses, reviews, case reports, guidelines, comments, letters, animal studies, academic dissertations, and books; (2) participants with other diseases, such as schizophrenia; (3) studies without a control group receiving sham stimulation; (4) participants received drug treatments; (5) missing data.

### 2.3 Quality assessment

**Figure 2:**
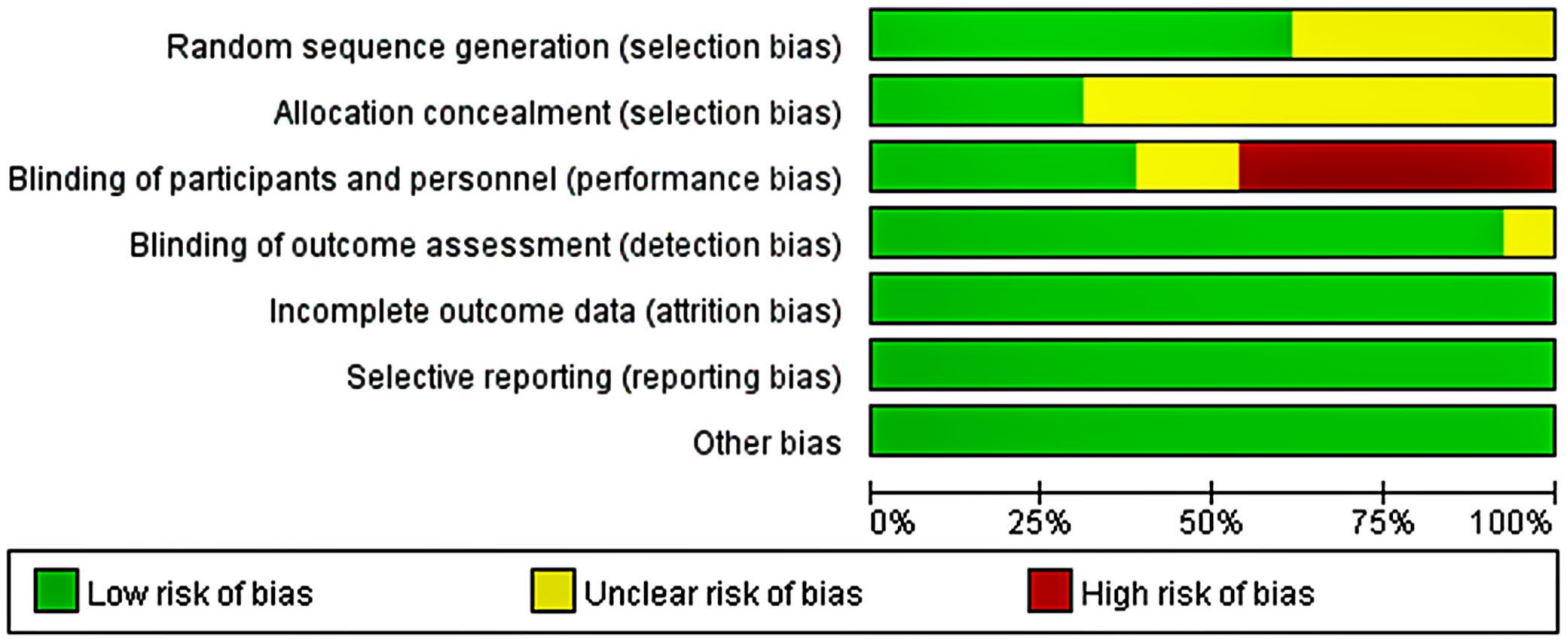
Risk of bias graph.

The quality of the included articles was assessed by two independent authors based on the Cochrane Collaboration tool. Seven domain biases were examined and the risk of bias for each domain was classified as low, high, or unclear (Figure 2). Any disagreement was discussed with and settled by the third arbitrator.

### 2.4 Data extraction

The data extracted from the selected studies included: (1) author(s), publication year, sample size; (2) diagnostic criteria; (3) intervention, stimulation site, number of stimulation sessions (treatment days/sessions of tDCS and rTMS), stimulation frequency (Hz of rTMS), stimulation intensity, duration, follow-up time, electrode and cognitive outcome measures.

### 2.5 Data analysis

All statistical analyses were performed by the Review Manager software (Review Manager 5.3). Memory functions were measured by different scales: the Rivermead Memory Behavioral Test (RMBT), Clinical Memory Scale (CMS), Episodic Memory Test, Digit Span Test-Backward (DST-B), Boston Naming Test, Delayed Matching to Sample (DMS) of the Neuropsychological Test Automated Battery (CANTAB), Auditory Verbal Learning Test (AVLT), Wechsler Memory Scale (WMS), and the Test de Aprendizaje Verbal Complutense (TAVEC). The Rivermead Memory Behavioral Test (RMBT) includes 14 subtests assessing aspects of visual, verbal, recall, recognition, immediate and delayed everyday memory (Zlomuzica et al., 2018). Clinical memory scale (CMS) is a memory function evaluation scale suitable for Chinese people and evaluates the auditory memory and visual memory (Zhang et al., 2017). Episodic Memory Test evaluates the episodic memory (Han et al., 2013). Digit Span Test-Backward (DSTB) assesses working memory capacity (Hibert et al., 2014). Boston Naming Test assesses verbal memory (Han et al., 2011; Gomes et al., 2019). Delayed Matching to Sample (DMS) of Cambridge Neuropsychological Test Automated Battery (CANTAB) evaluates the visual memory (Torgersen et al., 2012; Stonsaovapak et al., 2020). Auditory Verbal Learning Test (AVLT) evaluates immediate and delayed verbal memory (Zuo et al., 2018). Wechsler Memory Scale (WMS) scores are now derived for Older Adult Battery (65–90) and Adult Battery (16–69) and index include auditory memory, visual memory, visual working memory, immediate memory, and delayed memory (Han et al., 2011). And the Test de Aprendizaje Verbal Complutense (TAVEC) evaluates immediate memory (Benedet and Alejandre, 1998). They contained several aspects of memory, but all assessed the memory functions. In accordance with the Cochrane Handbook for Systematic Reviews, we calculated change values (mean ± standard deviation) from baseline to post-intervention as the outcomes of studies. We used the standardized mean difference (SMD) to assess the effect size of the interventions.

Heterogeneity was quantified by the I^2^ statistic; an I^2^ ≤ 25% suggested a low degree of heterogeneity, I^2^ ≤ 50% and > 25% indicated moderate heterogeneity. When meeting the two situations above, a fixed-effect model was used to integrate the results. I^2^ ≤ 75% and > 50% or I^2^ > 75% represented high or very high levels of heterogeneity, and a random-effect model was chosen. *p* < 0.05 was used to indicate a significant difference.

## 3 Results

### 3.1 Search results

Through the search of the eight databases, we obtained a total of 1749 records. Following the removal or duplicate records, 1,259 remained. Screening by title and abstract resulted in 43 articles. After reading the full text, we excluded the articles which not fit the content of this meta-analysis and articles without memory outcomes. Finally, we included 13 articles, including 486 MCI patients.

### 3.2 Characteristics of the included studies

The characteristics of the included articles are displayed in Table 1. Among the 13 studies, seven used rTMS and six used tDCS as the intervention. Eleven studies chose a single site of stimulation in the brain: eight studies stimulated the left DLPFC (F3) (Marra et al., 2012; Drumond Marra et al., 2015; Long et al., 2018; Wen et al., 2018; Gomes et al., 2019; Hu et al., 2020; Wang et al., 2021b; Satorres et al., 2023), one focused on the right DLPFC (F4) (Stonsaovapak et al., 2020), one study stimulated the left middle temporal gyrus (T3) (Gu et al., 2022), and one study stimulated the right cerebellum (Hu et al., 2016). The other two studies performed multi-site stimulation: one study focused on the bilateral DLPFC (F3, F4) (Han et al., 2013) and the other stimulated the bilateral frontal poles prefrontal area (Fp1, Fp2) and bilateral middle temporal gyrus (T3, T4) (Yan and Tao, 2011). All studies assessed memory functions as the outcomes following the treatment session immediately.

**Table 1:** Characteristics of the included studies.

No.	Study	Diagnostic criteria for MCI	Sample size (E/C)	Interventions	Site for stimulation	Number of stimulation sessions	Stimulation frequency, stimulation intensity	Duration (minutes)	Electrode	Follow-up time	Cognitive outcome measures
1	Yan and Tao (2011)	Petersen diagnostic criteria	12/13	rTMS	Bilateral frontal poles (Fp1, Fp2) Bilateral middle temporal gyrus (T3, T4)	800–1,000 pulses/day, once daily, 5 consecutive weekdays with interval of 1 month, totally 25 weekdays (sessions)	13 Hz, 100% RMT (Fp1, Fp2); 1 Hz, 80–120% RMT (T3, T4)	–	–	–	CMS (MQ): memory
2	Marra et al. (2012)	Subjective memory complaints and meeting clinical/neuropsychological criteria	9/10	rTMS	Left DLPFC (F3)	40 trains/day, once daily, 5 consecutive weekdays per week, 2 weeks, totally 10 weekdays (sessions)	10 Hz, 110% MT	–	–	Four weeks after treatment	RBMT: memory
3	Han et al. (2013)	Petersen diagnostic criteria	20/18	rTMS	Bilateral DLPFC (F3, F4)	600 pulses/day, once daily, 5 consecutive weekdays per week, 8 weeks, totally 40 weekdays (sessions)	20 Hz, 80% MT	–	–	–	Episodic memory test: episodic memory (long-term memory)
4	Drumond Marra et al. (2015)	Meeting clinical/neuropsychological criteria	15/19	rTMS	Left DLPFC (F3)	2000 pulses/day, once daily, 5 consecutive weekdays per week, 2 weeks, totally 10 weekdays (sessions)	10 Hz, 110% MT	–	–	One month after treatment	RBMT: memory
5	Long et al. (2018)	Petersen diagnostic criteria	15/15	rTMS	Left DLPFC (F3)	1000 pulses/day, once daily, 5 consecutive weekdays per week, 2 weeks, totally 10 weekdays (sessions)	15 Hz, 90% RMT	–	–	–	CMS (MQ): memory
6	Wen et al. (2018)	Petersen diagnostic criteria	23/22	rTMS	Left DLPFC (F3)	400 pulses/day, once daily, 5 consecutive weekdays per week, 4 weeks, totally 20 weekdays (sessions)	10 Hz, 80% RMT	–	–	One month after treatment	RBMT: memory
7	Wang et al. (2021b)	Guidelines for diagnosis and treatment of dementia and cognitive disorders in China	19/20	rTMS	Left DLPFC (F3)	1,500 pulses/day, once daily, 10 consecutive days (sessions)	10 Hz, 90% RMT	–	–	–	Auditory Verbal learning Test: immediate memory (working memory)
8	Hu et al. (2016)	Guidelines for diagnosis and treatment of dementia and cognitive disorders in China	18/21	tDCS	Right cerebellum	Once daily, 5 consecutive days (sessions)	–	20	1.2 mA: 35 cm^2^	–	WMS (Backwards Digit Span): working memory
9	Gomes et al. (2019)	Mayo clinic criteria	29/29	tDCS	Left DLPFC (F3)	Once daily, 2 days per week, totally 10 sessions	–	30	2 mA: 25 cm^2^	–	The Boston naming test: verbal memory (long-term memory)
10	Stonsaovapak et al. (2020)	The criteria of the MCI working group of the European Consortium on Alzheimer’s disease	23/22	tDCS	Right DLPFC (F4)	Once daily, 3 days per week, 4 weeks, totally 12 sessions	–	20	2 mA: 25 cm^2^	Eight weeks after treatment	CANTAB (DMS): visual memory (long-term memory)
11	Hu et al. (2020)	Meeting clinical/neuropsychological criteria	20/20	tDCS	Left DLPFC (F3)	Once daily, 5 days per week, 2 weeks, totally 10 days (sessions)	–	20	1 mA: 9 cm^2^	–	WMS (Backwards Digit Span): working memory
12	Gu et al. (2022)	Petersen diagnostic criteria and the diagnostic and statistical manual of mental disorders (DSM)	20/20	tDCS	Left middle temporal gyrus (T3)	Once daily, 5 days (sessions)	–	20	2 mA: 35 cm^2^	Four weeks after treatment	WMS (MQ): memory
13	Satorres et al. (2023)	Meeting clinical/neuropsychological criteria	17/16	tDCS	Left DLPFC (F3)	Once daily, 10 consecutive days (sessions)	–	20	2 mA: 25 cm^2^	–	TAVEC trial 1: immediate memory (working memory)

E/C, experimental group/control group; rTMS, repetitive transcranial magnetic stimulation; Hz, Hertz; RMT, resting motor threshold; CMS, Clinical Memory Scale; MQ, memory quotient; DLPFC, dorsolateral prefrontal cortex; MT, motor threshold; RMBT, the Rivermead Memory Behavioural Test; tDCS, transcranial direct current stimulation; WMS, Wechsler Memory Scale; CANTAB, Cambridge Neuropsychological Test Automated Battery; DMS, Delayed Matching to Sample; TAVEC, Test de Aprendizaje Verbal Complutense.

### 3.3 Heterogeneity analysis

To ensure that the included studies were statistically comparable, we examined all 13 articles and found that two studies (Hu et al., 2016, 2020) caused a high level of heterogeneity at 66% (*p* = 0.0004). When we removed them, total heterogeneity decreased to 22% (*p* = 0.23), resulting in low heterogeneity. The heterogeneity of tDCS subgroup changed from 84% (*p* < 0.0001) to 33% (*p* = 0.21), meaning that the degree of heterogeneity changed from very high to moderate.

In the study by Hu et al. (2016), the site of stimulation was the right cerebellum and the tDCS current intensity was 1.2 mA, while the other included articles targeted the cerebrum and the tDCS current intensity was 2 mA. In the study by Hu et al. (2020), at current intensity of 1.0 mA was used with a 9 cm^2^ electrode area. While the intensity of other studies was higher stimulation intensity (2 mA), which maybe mean the higher efficiency. Previous study revealed that anodal stimulation at 2 mA induced excitability enhancement compared to 1 mA anodal stimulation (Batsikadze et al., 2013). Studies have also shown that electrode size influences effects of tDCS and smaller electrodes size were more efficacious maybe due to the impact of more specific focal and less cross-network influence (Chase et al., 2020). These might be the reasons for high heterogeneity. Therefore, we removed these two studies from our statistical analysis to increase accuracy.

### 3.4 Meta-analysis in all protocols

**Figure 3:**
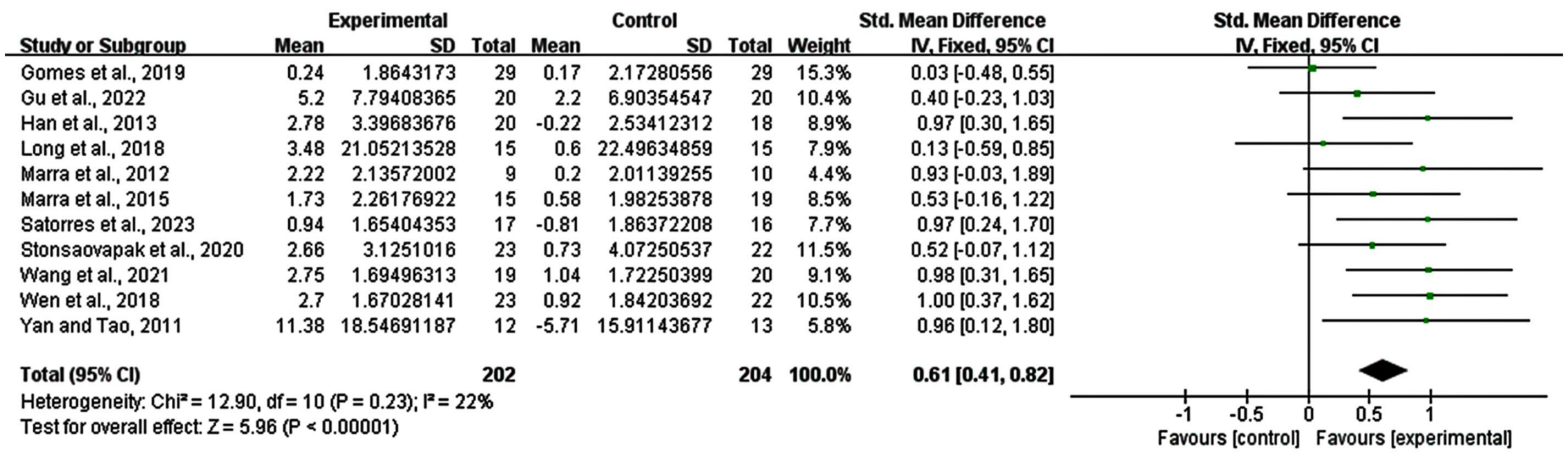
Meta-analysis of rTMS and tDCS on memory functions in MCI patients.

**Figure 4:**
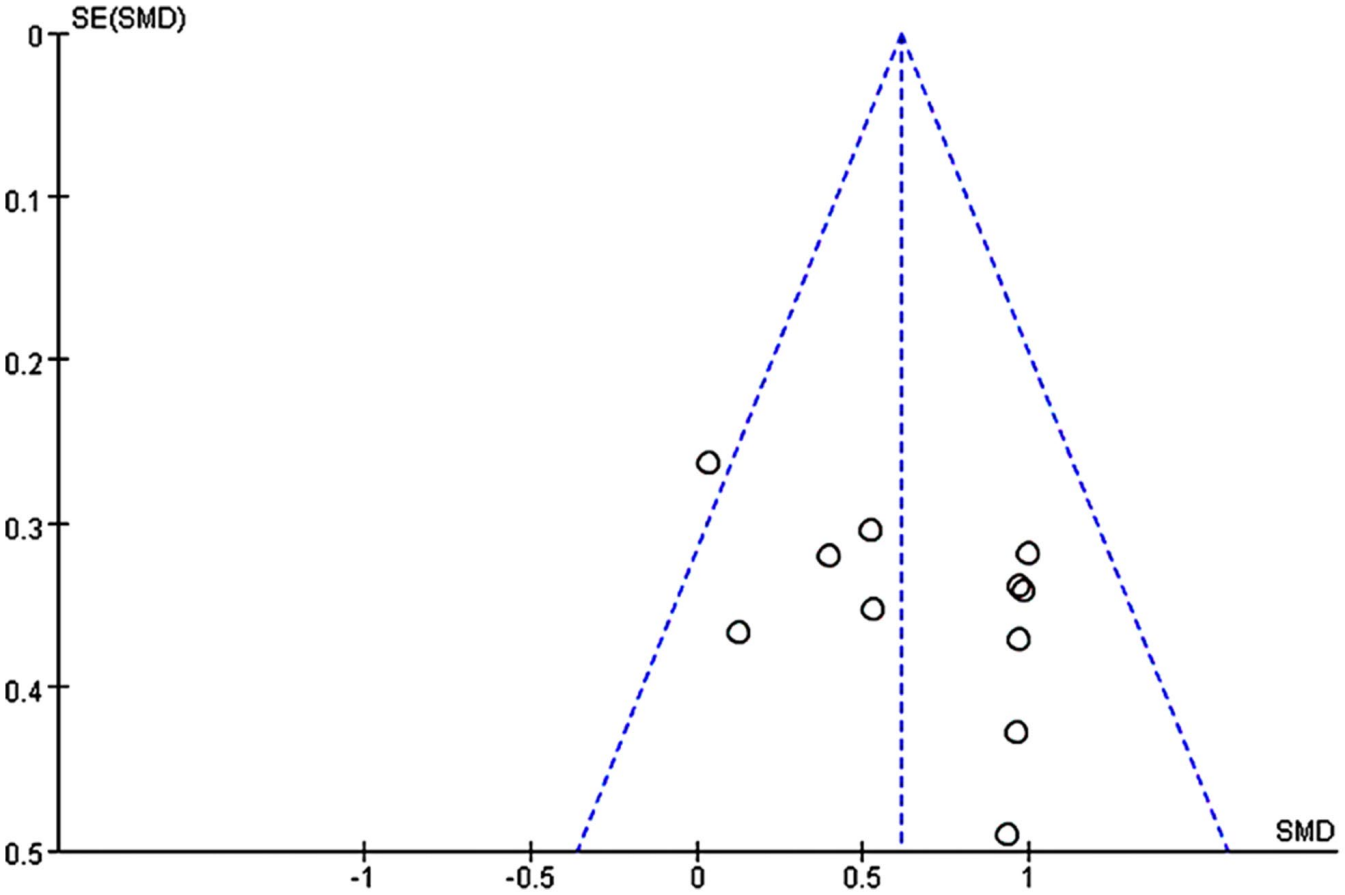
A funnel plot showing publication bias among included studies.

11 studies were included in the analysis of the whole heterogeneity (I^2^ = 22%, *p* = 0.23) (Figure 3) and analyzed via a fixed effect model. When comparing intervention-related changes of memory functions between the experimental and control groups, the SMD was 0.61 (95% confidence interval (CI): 0.41–0.82, *p* < 0.00001). The lack of marked asymmetry in the funnel plot as depicted in Figure 4 suggested there is no significant publication bias for results in this area.

### 3.5 Subgroup analysis: stimulation types

**Figure 5:**
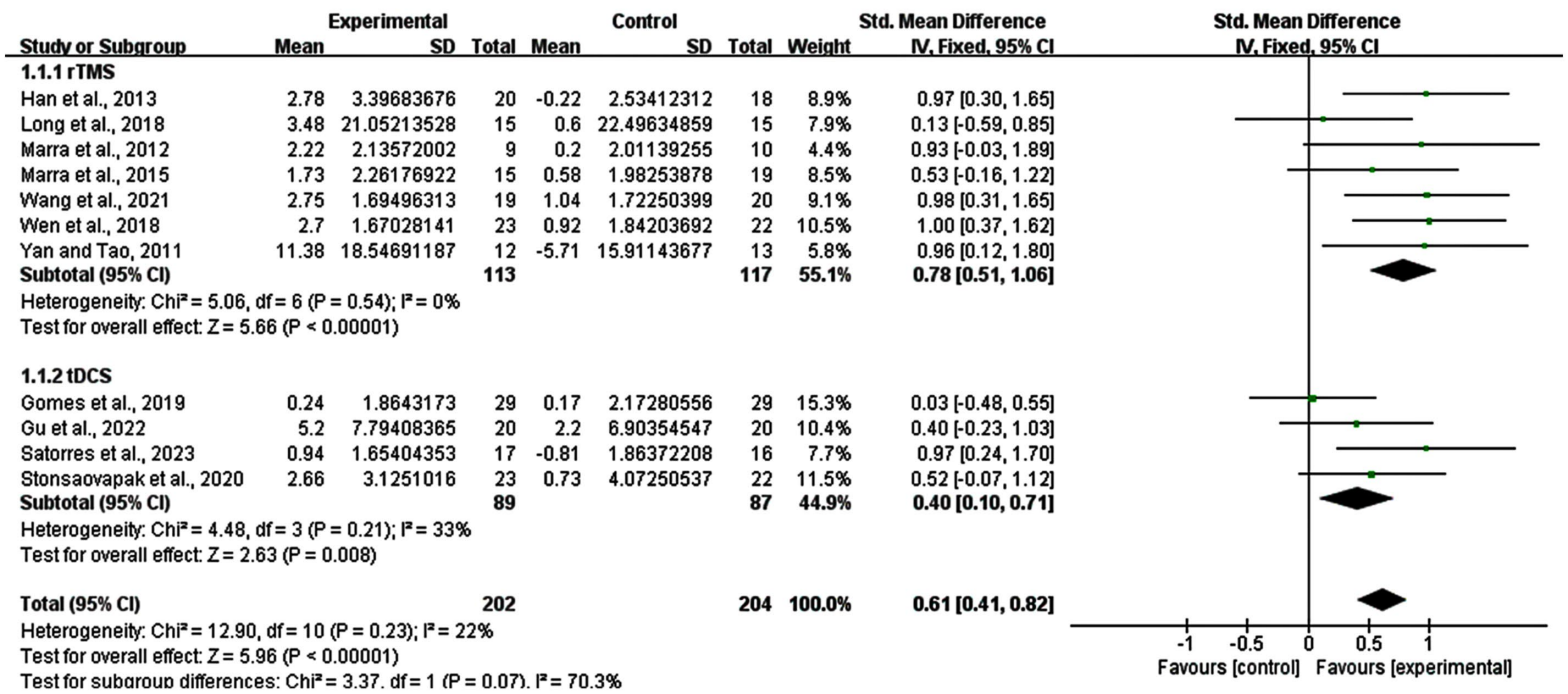
Subgroup analysis for stimulation types (rTMS vs. tDCS).

Subgroup analyses for the different stimulation types (tDCS, rTMS) were conducted. As depicted in Figure 5, a significant improvement of memory functions due to rTMS (SMD = 0.78; 95% CI: 0.51–1.06; *p* < 0.00001; I^2^ = 0%), as well as tDCS (SMD = 0.40; 95%CI: 0.10–0.71; *p* = 0.008; I^2^ = 33%) was revealed. The results showed furthermore that rTMS might have a larger improving effect on memory functions than tDCS.

### 3.6 Subgroup analysis: number of stimulation sessions

**Figure 6:**
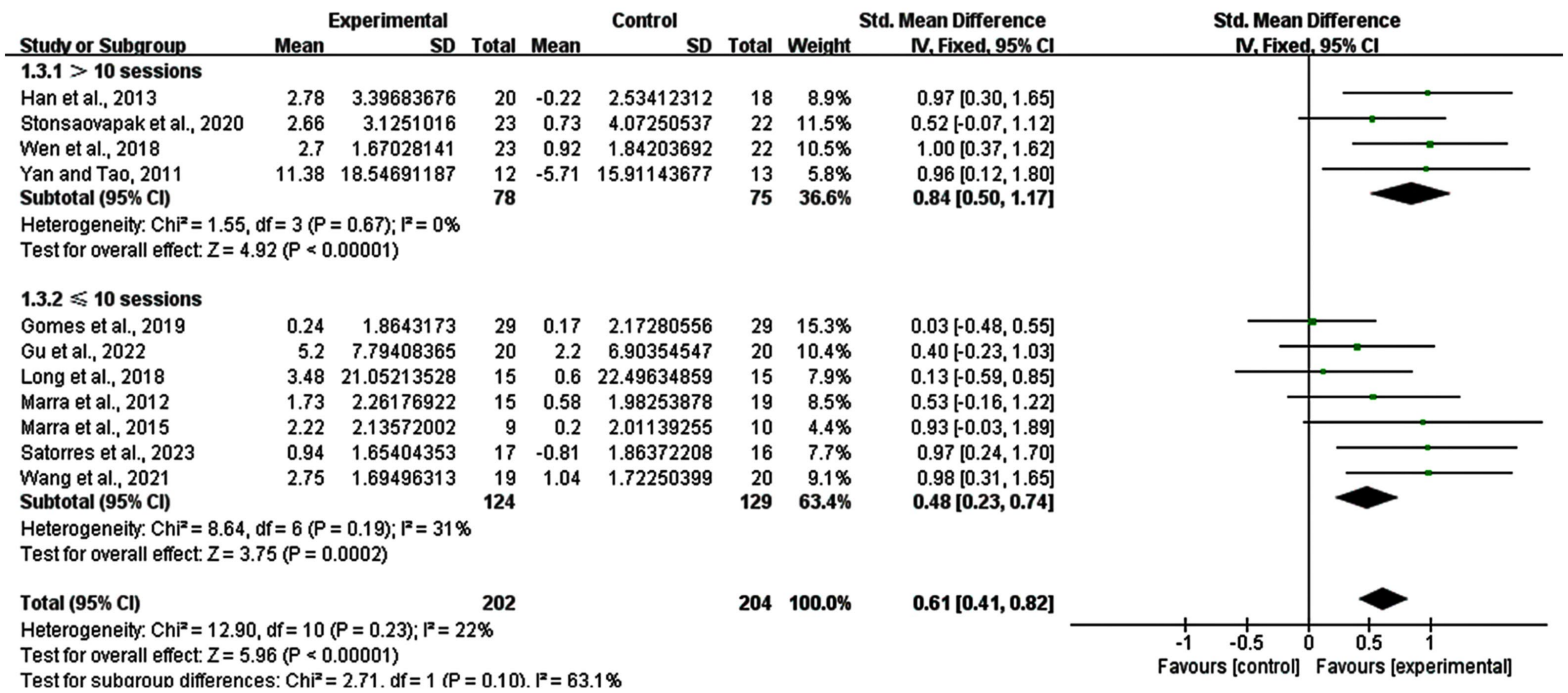
Subgroup analysis for number of stimulation sessions (> 10 sessions vs. ≤ 10 sessions).

The number of stimulation sessions differed from five sessions to 40 sessions. Therefore, we divided them into two groups (> 10 sessions and ≤ 10 sessions), analyzing the effects of the short-term and long-term. The result revealed that studies with >10 sessions had a SMD of 0.84 (95% CI: 0.50–1.17, *p* < 0.00001, I^2^ = 0%), while those with ≤10 sessions had a SMD of 0.48 (95% CI: 0.23–0.74, *p* = 0.0002, I^2^ = 31%) (Figure 6).

### 3.7 Subgroup analysis: number of stimulation sessions of rTMS

**Figure 7:**
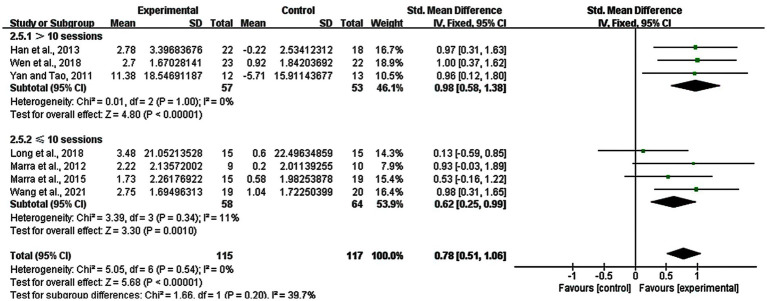
Subgroup analysis for number of stimulation sessions of rTMS (> 10 sessions vs. ≤ 10 sessions).

We also performed the subgroup analysis in the number of stimulation sessions only for rTMS, we explored the difference between long-term group (> 10 sessions) and short-term group (≤ 10 sessions) of rTMS. The result showed that there was a significant enhancement in the >10 sessions group (SMD = 0.98; 95% CI: 0.58–1.38; *p* < 0.00001; I^2^ = 0%) and the ≤10 sessions group (SMD = 0.62; 95% CI: 0.25–0.99; *p* = 0.0010; I^2^ = 11%) (Figure 7).

### 3.8 Subgroup analysis: stimulation site of rTMS

**Figure 8:**
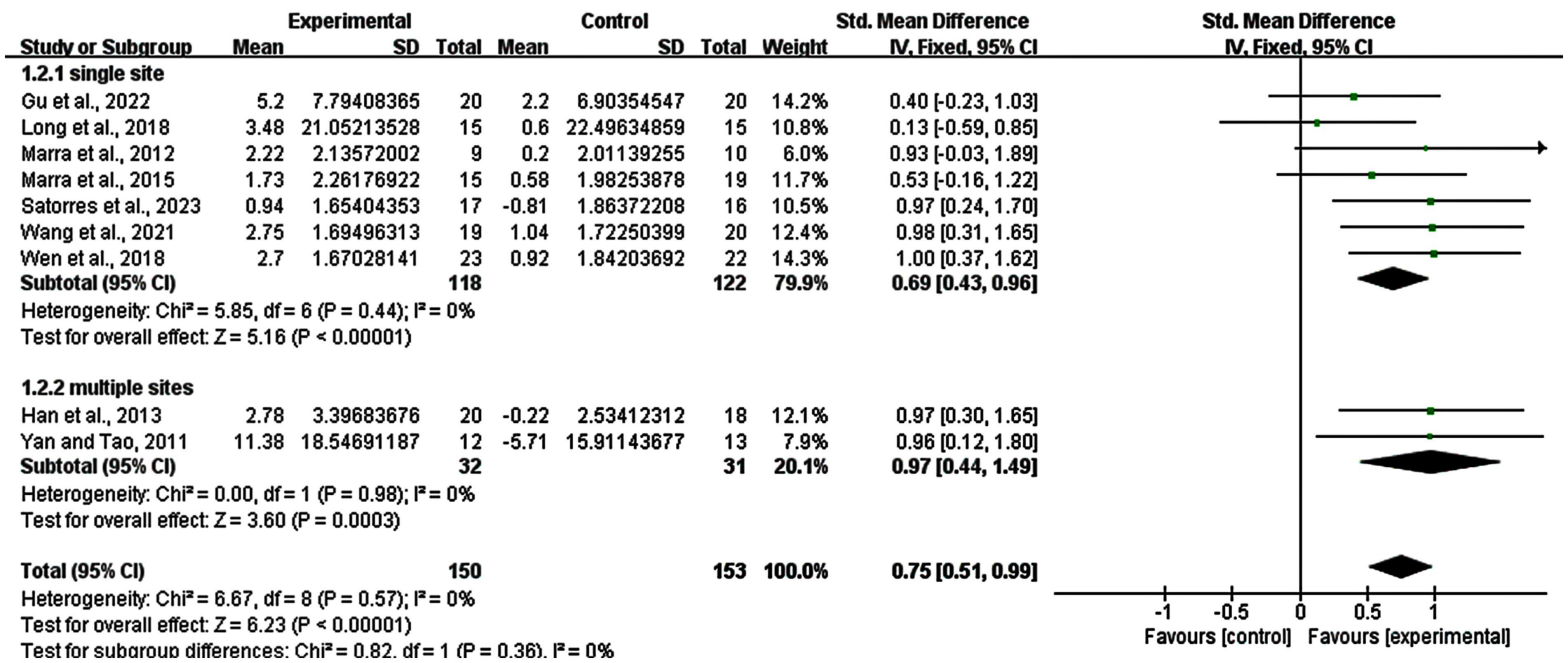
Subgroup analysis for stimulation site of rTMS (single site vs. multiple sites).

Regarding the subgroup analysis of the stimulation site, we explored the difference between single site and multiple sites of rTMS. Seven studies with 240 subjects involved used a single site and two studies with 63 subjects used multiple sites. The result showed that there was a significant enhancement in the single site group (SMD = 0.69; 95% CI: 0.43–0.96; *p* < 0.00001; I^2^ = 0%) and multiple sites group (SMD = 0.97; 95% CI: 0.44–1.49; *p* = 0.0003; I^2^ = 0%) (Figure 8).

### 3.9 Subgroup analysis: stimulation frequency of rTMS

**Figure 9:**
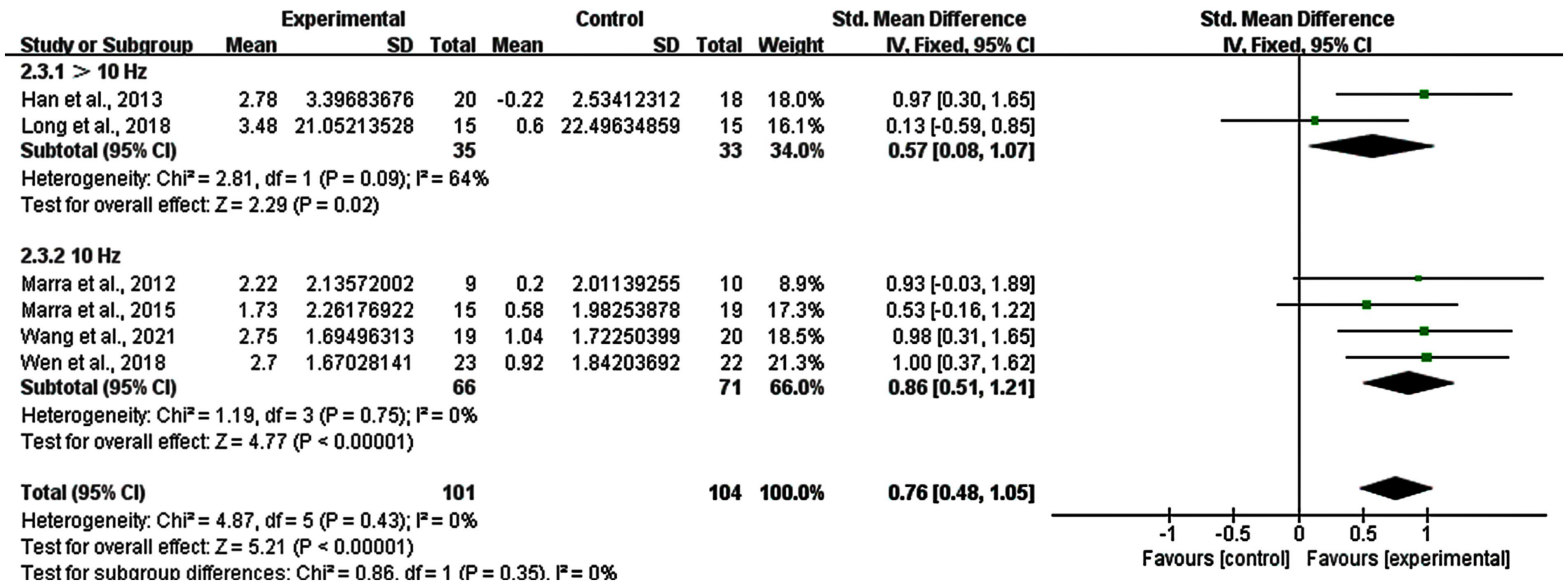
Subgroup analysis for stimulation frequency of rTMS (>10 Hz vs. 10 Hz).

Regarding the stimulation frequency of rTMS, it was classified in to two groups: >10 Hz and 10 Hz. We excluded one study using both 1 Hz and 13 Hz where it was difficult to determine the better frequency. Changes in the stimulation frequency of >10 Hz had a significant improvement (SMD = 0.57; 95% CI: 0.08–1.07; *p* = 0.02; I^2^ = 64%), as well as the 10 Hz group (SMD = 0.86; 95% CI: 0.51–1.21; *p* < 0.00001; I^2^ = 0%) (Figure 9).

### 3.10 Subgroup analysis: treatment persistent effects

**Figure 10:**
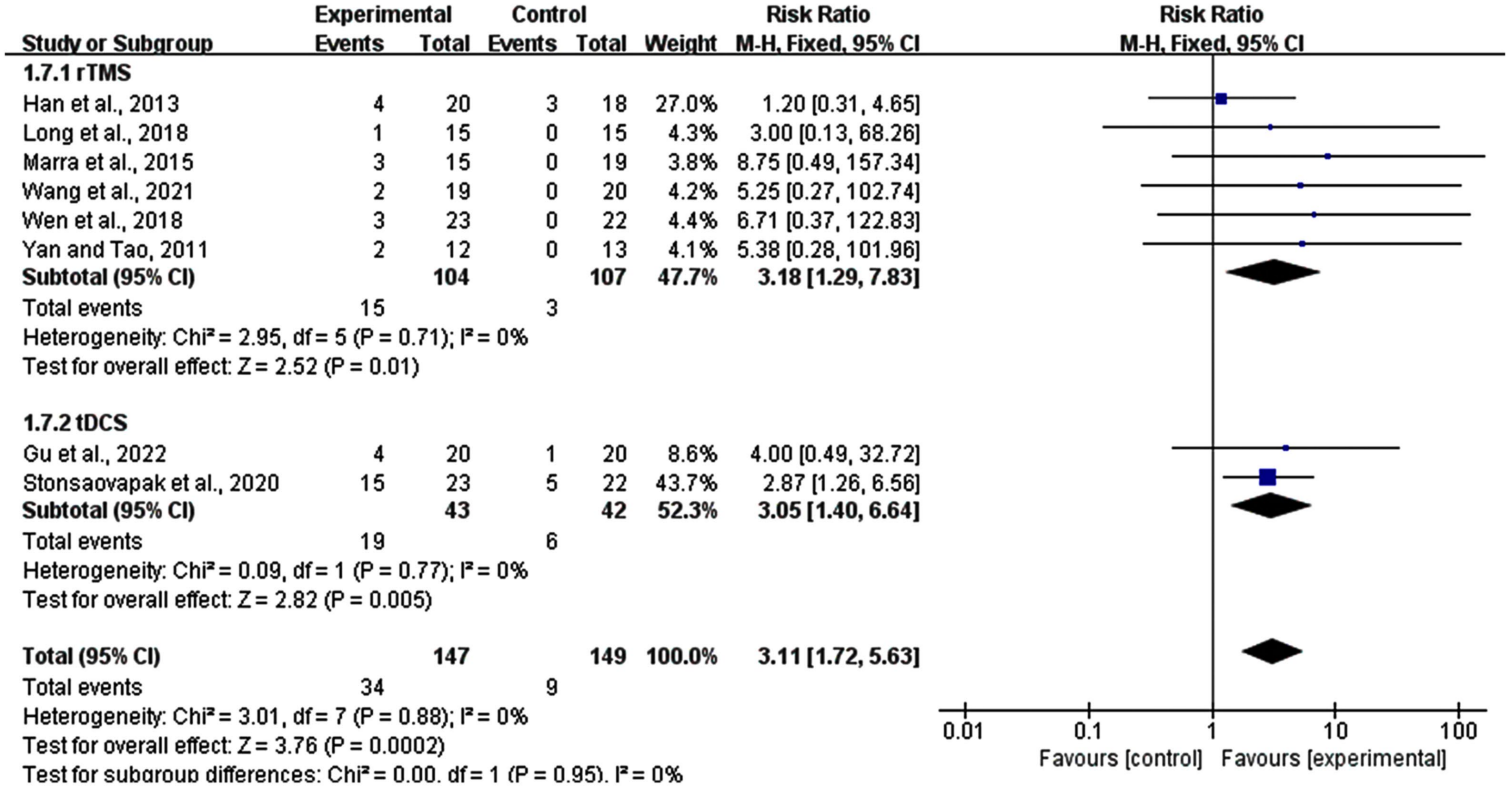
Subgroup analysis for treatment persistent effects (rTMS vs. tDCS).

A subgroup analysis based on the follow-up results of memory functions was also performed (five studies). Three studies of rTMS and two studies of tDCS reported the follow-up results. The real stimulation of rTMS and tDCS groups showed great persistent improvements in memory functions of MCI patients compared with sham stimulation group (SMD = 0.70; 95% CI: 0.40–1.00; *p* < 0.00001; I^2^ = 0%). And the result revealed that the subgroup of rTMS had a SMD of 0.93 (95% CI: 0.51–1.35, *p* < 0.0001, I^2^ = 0%), while the subgroup of tDCS had a SMD of 0.46 (95% CI: 0.03–0.89, *p* = 0.04, I^2^ = 0%) (Figure 10).

### 3.11 Subgroup analysis: adverse effects

**Figure 11:**
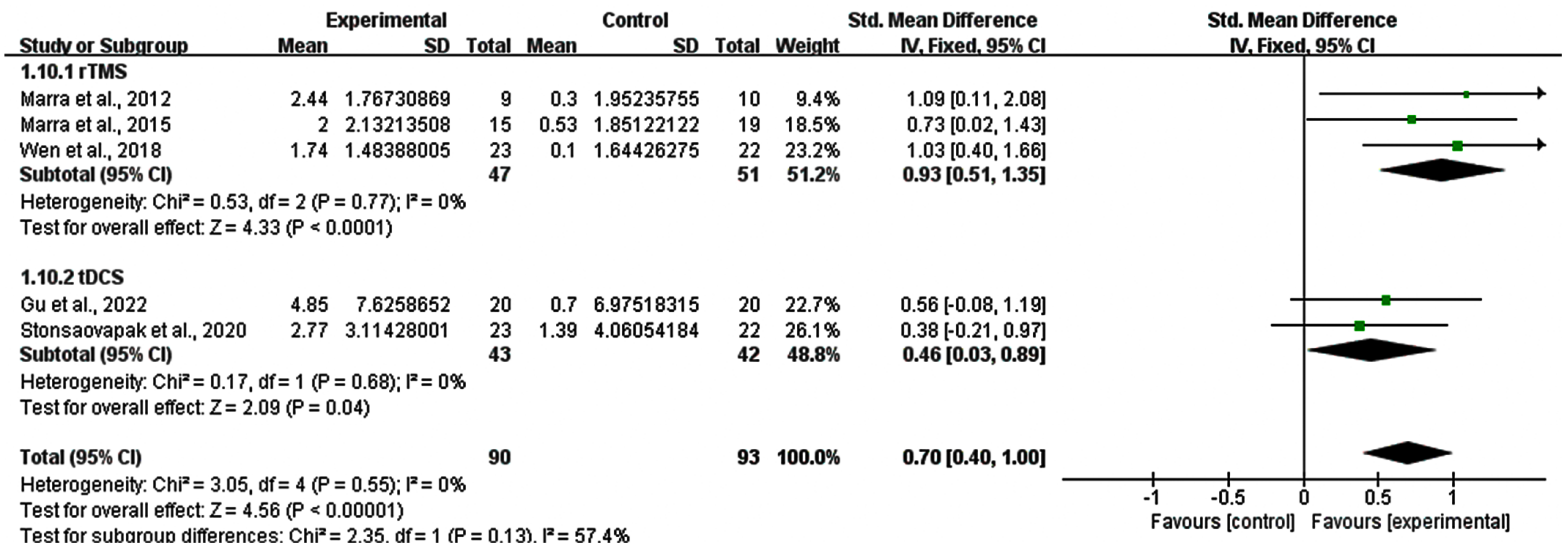
Subgroup analysis for adverse effects (rTMS vs. tDCS).

In this meta-analysis, eight studies reported adverse effects, including six rTMS studies and two tDCS studies. A total of 34 out of 147 participants in the experimental groups and nine out of 149 participants in sham stimulation groups reported discomfort during the procedure. The analyses revealed that compared to the sham stimulation group, adverse reactions were more likely in the real stimulation group (RR = 3.31, 95% CI: 1.72–5.63, *p* = 0.0002). In the subgroup of rTMS, 15 participants in experimental group and three participants in control group reported adverse effects, while in the tDCS subgroup, side effects were reported in 19, and 6 participants in the experimental and control groups, respectively. rTMS was slightly more likely to appear side effects (risk ratio (RR) = 3.18, 95% CI: 1.29–7.83, *p* = 0.01) than tDCS (RR = 3.05, 95% CI: 1.40–6.64, *p* = 0.005). Most of the patients reported temporary mild headache, tingling sensations, or dizziness. Also skin itching (two persons), skin redness (one person), and fatigue (two person) were reported. All these symptoms were tolerable and recovered after experiments (Figure 11).

## 4 Discussion

To the best of our knowledge, this is the first meta-analysis to explore the effects of both, rTMS and tDCS on memory functions in MCI patients. This meta-analysis aimed to assess the efficacy of rTMS and tDCS in improving memory performance in MCI patients. 11 articles with 406 MCI patients were analyzed, while two studies were removed due to high heterogeneity. The results suggest that both rTMS and tDCS improved memory functions in patients with MCI compared with sham stimulation, but the efficacy of the interventions, different stimulation sites, frequencies, and number of stimulation sessions differed. Furthermore, we also explored the adverse effects in rTMS group and tDCS group.

A couple of previous meta-analyses conducted subgroup analyses for rTMS and tDCS in related fields. Teselink et al. explored the effects of rTMS and tDCS on global cognition and neuropsychiatric symptoms in Alzheimer’s Disease (AD) /MCI. In that study, subgroup analyses revealed positive effects of rTMS, but not tDCS (Teselink et al., 2021). Wang et al. explored behavioral and psychological symptoms of dementia before and after rTMS and tDCS in AD patients, which also revealed that rTMS significantly alleviated respective symptoms (Wang et al., 2020b). In addition, subgroup analyses on rTMS and tDCS in meta-analyses of other diseases also found a similar result pattern in other diseases, such as poststroke dysphagia, gait speed after stroke, and spinal cord injury (Yang et al., 2015; Vaz et al., 2019; Yu et al., 2020; Wang et al., 2021a). The subgroup analysis conducted for rTMS and tDCS in the present meta-analysis showed that both intervention tools had significant effects on improving memory functions in patients with MCI, but that rTMS was more efficient than tDCS. While several intervention parameters, which differed between studies, might affect stimulation outcomes, such as the targeted region, and number of stimulation sessions (Prehn and Flöel, 2015; Lefaucheur et al., 2020), also other factors might contribute, including mechanistic ones. rTMS evokes action potentials by influencing the strength of glutamatergic synapses and inducing suprathreshold depolarization of neuronal membranes (Gomes-Osman et al., 2018; Polanía et al., 2018). tDCS trigger s activation of voltage-gated pre-and postsynaptic sodium and calcium channels through subthreshold depolarization, and will increase the presynaptic release of excitatory transmitters as well as the postsynaptic calcium influx and then cause alterations of resting membrane potential (Nitsche and Paulus, 2001; Fertonani and Miniussi, 2017; Stagg et al., 2018). This meta-analysis included lower studies using tDCS than rTMS improving memory functions of patients with MCI, which could have also influenced the statistical result because small trails lack power and false positives may occur (Pocock and Stone, 2016). Therefore, more studies are required to explore the effects of tDCS on memory performance in MCI patients.

The results also showed that rTMS targeted on both a single site and multiple sites enhanced memory functions among MCI. It was not possible to make conclusion about targeted region of tDCS in this meta-analysis. Furthermore, we found that stimulating multiple sites were more efficient than a single site. This was similar to the results of previous studies. Lin et al. and Wang et al. reported that cognitive enhancement following rTMS over multiple sites was superior to single site stimulation (Lin et al., 2019; Wang et al., 2020a). Liao et al. (2015) found that the effect of rTMS over the bilateral DLPFC was better than that of only left DLPFC stimulation. In this meta-analysis, two rTMS studies targeted multiple sites, and seven rTMS studies focused on a single site. Specifically, multiple site stimulation was conducted over the bilateral DLPFC (F3, F4), the bilateral frontal poles (Fp1, Fp2) as well as the bilateral middle temporal gyrus (T3, T4), and single site stimulation was conducted over the left DLPFC (F3). Cognitive neuroscience has proven the involvement of the PFC in human memory, attention, perception via top-down signals to control various cognitive processes (Tanigawa et al., 2022). The DLPFC is a core area of cognitive functions and has extensive connections with other brain regions. Barbey et al. drew many brain-injured (significant damage to left and/or right DLPFC) and neurologically healthy Vietnam veterans to explored the necessity of DLPFC for working memory, and deficiency was observed in the brain-injured patients group for working memory (Barbey et al., 2013). This indicated that working memory was mainly processed in the DLPFC. It may also interact with the medial temporal network, contributing to executive and memory function (Blumenfeld and Ranganath, 2006; Blumenfeld et al., 2011; Chou et al., 2020). A study revealed that patients with left or right medial temporal lobe resection had difficulties in retrieving autobiographical memories (Noulhiane et al., 2007; Tanigawa et al., 2022). Therefore, rTMS and tDCS targeted on the DLPFC might improve the working memory in neurological and psychiatric disorders, such as MCI (Fox et al., 2014). Studies have shown varying degrees of success regarding the therapeutic effects by targeting two sites, which greatly increases the stimulation volume (Rossi et al., 2009).

In addition, both the short-term (≤ 10 sessions) and long-term (> 10 sessions) stimulation effects were significantly improved the memory function of MCI patients, with the long-term effect showing greater benefits. Furthermore, we found that long-term rTMS interventions was better than short-term interventions in improving memory performance in MCI patients. These results were consistent with the findings of previous meta-analyses, long-term effects of rTMS showed greater benefits than short-term interventions in improving cognitive functions of patients with MCI/AD (Lin et al., 2019; Wang et al., 2020a; Zhang et al., 2021). The study by Wang et al. (2020a) compared the effects of long-term treatment (> 10 sessions) and short-term treatment (≤ 10 sessions) for cognitive function of rTMS in patients with AD, and their results showed that long-term rTMS had longer aftereffects. The meta-analysis by Lin et al. found that the effects of long-term treatment may be confounded with stimulating multiple sites, which resulted in better effects on improving memory functions in patients with AD (Lin et al., 2019). Due to the few studies available, further studies should explore the specific contribution of respective factors.

High-frequency (≥ 5 Hz) stimulation raises cortical excitability, with low frequency (≤ 1 Hz) doing the opposite (Cirillo et al., 2017). Early research has proven that 20 Hz can increase cortical excitability while 1 Hz decreased excitability (Gangitano et al., 2002). Ahmed et al. (2012) performed a comparison between 20 Hz and 1 Hz rTMS in cognitive functions of patients with AD, indicating that higher frequency rTMS was more useful. In our subgroup analysis of rTMS stimulation frequency, there were seven studies: one trial applied 20 Hz rTMS, one trial used 15 Hz rTMS, four trails used 10 Hz rTMS, and one trial used both 13 Hz and 1 Hz rTMS; we removed the last one due to the mix of high-frequency (≥ 5 Hz) and low frequency (≤ 1 Hz), and sorted the patients into two groups: > 10 Hz and 10 Hz. In summary, 10 Hz was more effective than >10 Hz. Therefore, 10 Hz rTMS showed a better improvement on memory functions in patients with MCI than 15 Hz and 20 Hz. However, this result was inconsistent with the conclusion of previous findings, such as the study by Wang et al. (2020a), which reported that 20 Hz stimulation resulted in better cognitive function than 10 Hz and 1 Hz rTMS. This difference might be due to the lack of 20 Hz rTMS studies included in this meta-analysis. Therefore, the result should be interpreted with caution. A larger sample of high-quality studies is required to explore this conclusion.

In addition, the stimulation duration of tDCS is also an important factor. Most studies used tDCS with duration of 20 min or 30 min, which showed good effects in improving the neurological functions in MCI or AD patients. In included studies, there were three studies using 20 min and one study using 30 min. However, it appeared that tDCS with duration of at least 20 min was required to induce improvement of memory functions in patients with MCI. Within certain limits, a longer stimulation duration may enhance the efficacy of the stimulation effects, but, prolonged excitation may eventually switch to inhibition (Monte-Silva et al., 2013; Stagg et al., 2018). Thus, it still needs more studies to explore the fit duration of tDCS.

How long the stimulation effects persist is a crucial aspect. We collected follow-up memory functions results of five included studies, with three studies of rTMS and two studies of tDCS. The results showed that real stimulation of rTMS and tDCS existed persistent effects in the fourth weeks, eighth weeks or one month after treatment. These results might relate to the feature of long-lasting cortical excitability elevations. rTMS induced long-lasting changes beyond the stimulation period in human brain activity, which might through removing GABAergic inhibition by transient deafferentation (Nitsche and Paulus, 2001; Klomjai et al., 2015). tDCS can also prolong the excitability of human brain activities in synaptic efficacy by increasing postsynaptic calcium influx (Nitsche and Paulus, 2001). The treatment persistence effects are critical for clinical practitioners to understand the expected timeline for treatment outcomes and to provide patients with informed guidance on when they might begin to experience the benefits of the treatment.

Overall, this meta-analysis revealed that rTMS and tDCS are safe and effective methods for improving memory functions in MCI patients. The outcome showed that adverse effects were more likely appeared in real stimulation group rather than sham stimulation group. And rTMS was more likely to appear than tDCS, which might because the studies (two) and participants (43 in experimental group and 42 in control group) of tDCS group were lower than rTMS group (six studies with 104 participants in experimental group and 107 participants in control group), while previous study considered that trails with small sample capacity lack power and false positives may occur (Pocock and Stone, 2016). The result revealed that adverse reactions were more likely in the experimental group, but almost all participants could tolerate the stimulation. Some mild adverse effects were reported, such as brief tingling, itching sensation, skin redness, mild headache, dizziness, and fatigue during the experiment. All symptoms were alleviated within 1 to 2 h.

The motor threshold is the most relevant parameter of TMS, which is used to determine the intensity of rTMS. It consists of the resting motor threshold and active motor threshold (Gomes-Osman et al., 2018). In most studies, the stimulation intensity was lower than 130% of the resting motor threshold to ensure safety (Rossi et al., 2009). However, due to interference factors of drugs during stimulation, underlying pathological factors, and other physiological reasons, no consensus on the stimulation intensity of rTMS has been reached in previous studies (Rossi et al., 2009). Thus, further research should explore the parameter of stimulation intensity of rTMS.

This meta-analysis had some limitations. First, we did not include data missing articles or studies written in other languages except for English or Chinese. Second, the measurement scales of memory functions were different between the studies because of the lack of studies, which assessed the several aspects of memory functions. Thus, we used the SMD to synthesize the effect size to solve this as far as possible. Third, we did not compare the targeted areas one by one to find the best stimulation region for memory functions improvement in MCI patients because the number of studies of each subgroup should include at least two studies. We only included rTMS studies in the subgroup analysis of stimulation sites and we could not make conclusion on stimulation site of tDCS. Fourth, we did not include the studies that using TMS and tDCS combined with other interventions, which is an excellent research question for the future. At last, the number of tDCS studies was relatively small and there was no enough data to do subgroup analyses so that we could not make a conclusion of the parameters of tDCS. Therefore, more researches are required to validate the present findings in stimulation regions, number of stimulation sessions, frequencies and intensities of rTMS as well as durations of tDCS to overcome the knowledge gaps.

## 5 Conclusion

The results of this review and meta-analysis suggest that rTMS and tDCS are safe and effective in improving memory functions in MCI patients and rTMS showed better effects than tDCS. rTMS targeted on multiple sites with a frequency of 10 Hz over 10 sessions seemed to show the greatest effect. We could not conclude parameters of tDCS due to insufficient data. The analysis showed knowledge gaps to overcome to optimize interventions. This result might facilitate the progress in improving the memory functions in patients with MCI.

## Data availability statement

The original contributions presented in the study are included in the article/supplementary material, further inquiries can be directed to the corresponding authors.

## Author contributions

MH: Data curation, Formal analysis, Writing – original draft. MN: Writing – review & editing, Visualization. YL: Writing – review & editing, Data curation. HH: Writing – review & editing, Visualization. XL: Conceptualization, Visualization, Writing – review & editing. FQ: Conceptualization, Visualization, Writing – review & editing.
